# The effect of timing of physical exercise on glycemia: a systematic review and meta-analysis of human intervention studies

**DOI:** 10.1007/s40200-026-01954-z

**Published:** 2026-05-29

**Authors:** Samantha B.J. Schipper, Romy Slebe, Linda J. Schoonmade, Denis P. Blondin, David J.T. Campbell, André C. Carpentier, Jean-Pierre P. Després, Joris Hoeks, Andries Kalsbeek, Parminder Raina, Patrick Schrauwen, Mireille J. Serlie, Dirk Jan Stenvers, Chun-Xia Yi, Renée de Mutsert, Joline W.J. Beulens, Femke Rutters

**Affiliations:** 1 Department of Epidemiology and Data Science, Amsterdam Public Health Research Institute, Amsterdam UMC, location VUmc, Amsterdam, The Netherlands; 2 Amsterdam Public Health, Health Behaviours & Chronic Diseases, Amsterdam, The Netherlands; 3 University Library, Vrije Universiteit Amsterdam, Amsterdam, The Netherlands; 4 Centre de recherche du Centre hospitalier universitaire de Sherbrooke, Sherbrooke, QC J1H 5H3 Canada; 5 Department of Medicine, Division of Neurology, Faculty of Medicine and Health Sciences, Université de Sherbrooke, Sherbrooke, QC J1H 5H3 Canada; 6 Department of Medicine, University of Calgary Cumming School of Medicine, Calgary, AB Canada; 7 Department of Community Health Sciences, University of Calgary Cumming School of Medicine, Calgary, AB Canada; 8 Department of Cardiac Sciences, University of Calgary Cumming School of Medicine, Calgary, AB Canada; 9 Department of Medicine, Division of Endocrinology, Faculty of medicine and Health Sciences, Université de Sherbrooke, Sherbrooke, QC J1H 5H3 Canada; 10 Department of Kinesiology, Université Laval and Centre de recherche sur les soins et les services de première ligne, Québec City, QC Canada; 11 Department of Nutrition and Movement Sciences, NUTRIM Institute of Nutrition and Translational Research in Metabolism, Maastricht University, Maastricht, The Netherlands; 12 Department of Endocrinology and Metabolism, Amsterdam UMC, University of Amsterdam, Amsterdam, the Netherlands; 13 Amsterdam Gastroenterology Endocrinology Metabolism, Amsterdam, The Netherlands; 14 Netherlands Institute for Neuroscience (NIN), an Institute of the Royal Netherlands Academy of Arts and Sciences (KNAW), Meibergdreef 47, Amsterdam, 1105BA The Netherlands; 15 Department of Health Research Methods, Evidence, and Impact, McMaster University, Hamilton, ON Canada; 16 McMaster Institute for Research on Aging, McMaster University, Hamilton, ON Canada; 17 Institute for Clinical Diabetology, German Diabetes Center, Leibniz Institute for Diabetes Research at Heinrich Heine University Düsseldorf, Düsseldorf, Germany; 18 German Center for Diabetes Research (DZD), Partner Düsseldorf, Neuherberg, Germany; 19 Clinical Epidemiology, Leiden University Medical Center, Leiden, The Netherlands; 20 Yale School of Medicine, Section of Endocrinology, New Haven, USA; 21 Julius Centre for Health Sciences and Primary Care, University Medical Centre Utrecht, Utrecht, the Netherlands; 22 Epidemiology and Data Science, Amsterdam UMC, location Vrije Universiteit Medisch Centrum, Van der Boechorststraat 7, 1081BT, Amsterdam, Netherlands

**Keywords:** Physical exercise, Circadian dysregulation, Circadian clocks, Glycemic control, Glucose metabolism

## Abstract

**Aim:**

Physical exercise (PE) may be important for glucose metabolism. Therefore, this systematic review and meta-analysis aims to investigate the effect of PE performed in the morning versus afternoon or evening on glycemia parameters in human intervention studies.

**Methods:**

MEDLINE and Embase.com were searched until February 2023 for intervention studies in the general adult population, examining the effect the timing of PE on glycemia parameters after one or multiple bouts of exercise. Results were meta-analysed using a random-effects models where appropriate or were described using qualitative synthesis.

**Results:**

55,569 publications were screened for a series of reviews, of which 20 studies (680 participants) were included in this review. In studies including one bout of PE (*n* = 12), glucose levels measured directly after PE were 0.26 mmol/L (95%CI -0.18;0.70, I^2^ = 81%, *n* = 3) higher after morning PE, compared to afternoon PE. Studies including repeated PE for 1–12 weeks (*n* = 8), showed that fasting glucose was 0.25mmol/L (95%CI 0.02;0.47, I^2^ = 32%, *n* = 7) higher in morning PE, compared to afternoon/evening PE. Other outcomes (e.g. 24 h mean glucose, HOMA-IR) could not be meta-analyzed and were assessed qualitatively, showing no associations or results in line with the results above. Only time in range (TIR) and hypoglycemic events tended to favor morning PE.

**Conclusion:**

This review suggests that fasting glucose may be higher following morning PE, compared with afternoon or evening PE in long-term studies. Evidence on glucose directly after PE in acute studies and other glycemic outcomes remains limited and inconclusive. The included studies were generally small, heterogeneous in design and interventions, and often did not account for important factors such as nutritional intake and chronotype. Therefore, well-designed, adequately powered trials that account for lifestyle factors and assess clinically relevant outcomes are needed to clarify these associations.

**Trial registration:**

This review is part of a larger search ‘The effect of altering timing of physical activity, sleep and energy intake on glycemia and Type 2 Diabetes risk in humans’, of which the protocol was registered in the PROSPERO database on November 27^th^, 2021 under number: CRD42021287828.

**Supplementary Information:**

The online version contains supplementary material available at 10.1007/s40200-026-01954-z.

## Introduction

The rapidly increasing prevalence of type 2 diabetes (T2D) and its associated complications pose a serious global health burden [1]. Increasing physical exercise (PE), among other behavior interventions, is a widely accepted strategy for the management and prevention of this metabolic disorder [2]. PE has beneficial effects on glycemic control and insulin sensitivity and is associated with a reduced incidence of diabetes-related complications, including cardiovascular disease and mortality [3].

Recently, circadian misalignment between the endogenous circadian system and behavioral cycles (e.g. feeding-fasting, sleep-wake, activity-rest) has been identified as potential risk factor for T2D [4]. The circadian system produces daily rhythms in various physiological processes, including insulin sensitivity and glucose metabolism [5]. For instance, it is known that glucose tolerance decreases from morning to evening as a result of a gradual decrease of insulin sensitivity and pancreatic β-cell function over the day in people without diabetes [6]. However, among people with diabetes, this rhythm may be reversed with lower insulin sensitivity in the morning [7]. These rhythms are produced by a central pacemaker in the suprachiasmatic nucleus of the hypothalamus and peripheral oscillators in most peripheral tissues that can respond to external environmental stimuli (e.g. light-dark) and behavioral cycles [5, 8]. Therefore, performing PE at a moment aligned with the peripheral muscle clock could play an important role in improving parameters related to glucose metabolism and glycemic control.

Several studies have investigated the effect of timing of PE on glycemia parameters in T2D (e.g [9, 10]), and two previous systematic reviews, including one meta-analysis, have investigated the time of day-dependent effect of PE on cardiovascular risk factors [11, 12]. These studies only evaluated blood glucose as glycemia parameter for which no significant difference between morning PE and afternoon PE was found. Moreover, glycemia may not be a very sensitive measure of cardiovascular risk, which emphasizes the need to recognize and investigate outcomes of disease progression in (pre)diabetes such as HbA1c and insulin sensitivity. Therefore, this systematic review and meta-analysis aimed to investigate the effects of PE performed in the morning versus PE performed in the afternoon or evening on a multitude of indices related to blood glucose homeostasis in human intervention studies.

## Materials and methods

### Data sources and search strategy

We conducted a systematic review and meta-analysis in accordance with the Preferred Reporting Items for Systematic Reviews and Meta-Analyses (PRISMA) guidelines [13]. This review was part of a series of systematic reviews and meta-analyses, of which the protocol was registered in the PROSPERO database under number CRD42021287828 on November 27, 2021. Clinical trial number: not applicable. Human Ethics and Consent to Participate declarations: not applicable. This series investigates the effect of altering timing of PE, sleep and energy intake on glycemia in human intervention trials [14, 15]. The current review focuses on the timing of PE.

A search using Medline via Ovid and Embase.com from inception until February 24, 2023 was conducted with the help of a medical librarian (LS). Furthermore, reference lists of the included publications were screened to identify any additional relevant publications. A combination of thesaurus terms and free text terms related to interventions on timed lifestyle behavior change and outcomes of interest was used: e.g. circadian rhythms, incident type 2 diabetes, prediabetes, glycemia; and glycemia parameters, such as HbA1c, fasting plasma glucose, fasting insulin and two-hour glucose area under the curve (AUC). The search was restricted to clinical trials involving adult human participants. A full overview of the search strategy is provided in Appendix S1.

### Study selection

Publications were included if (1) the design was quantitative and reported on controlled trials that were either randomized, non-randomized, or crossover; (2) the publication was written in English, French or Dutch languages; (3) the full text was available; (4) the population consisted of human adults (≥ 18 years old), with no exclusions on health status; and (5) the study measured at least one of the outcomes of interest, namely: type 2 diabetes incidence, prediabetes as defined by the World Health Organization (WHO) or American Diabetes Association (ADA) [16, 17], which could only be included if long-term trials are available; or glycemia parameters (e.g. HbA1c, fasting plasma glucose, fasting insulin, glucose AUC). Outcomes were collected from medical records, biomarker data, glucometer/sensor data, validated surveys and/or questionnaires. Blood glucose was considered the primary outcome in acute studies and overnight fasting glucose was the primary outcome in long-term studies. All other reported glycemia parameters were considered secondary outcomes. Articles were excluded if (1) blood glucose was measured within 3 h after energy intake, arbitrarily based on average postprandial blood glucose response either in one or both study arms; or (2) morning PE was not the same intervention (duration, type of PE, etc.) as afternoon or evening PE. No publications were excluded based on type or duration of PE. Instead, sensitivity analyses were performed accordingly. Furthermore, studies measuring response either directly after one bout of PE (acute studies) or after a longer period of time after multiple bouts of PE (long-term studies) were included. When study results were reported more than once, only the most recent publication was included.

### Study screening and data extraction

The title and abstract of all retrieved publications were screened using ASReview (version 0.18), an open-source machine learning-aided program that contributes to a less error-prone and more efficient screening process [18, 19]. During title and abstract screening, the program iteratively rearranged the publications based on relevance, learning from the reviewer’s choices [18, 19]. This screening process was performed by one reviewer (FR) until more than 300 consecutive publications were excluded. The process was manually checked by two reviewers (SS and RS) through screening all publications the first reviewer assigned as potentially relevant and a random sample (± 200 titles and abstracts) of all the titles and abstracts excluded by ASReview. Subsequently, full text versions of potentially eligible publications were retrieved (if necessary, by contacting one of the authors), assessed according to the inclusion and exclusion criteria by two independent reviewers (SS and RS), and reasons for exclusion were recorded. The initial positive and negative agreement proportion were 75.5% and 87.6%, respectively. Discrepancies were resolved through discussion or involvement of a third reviewer (FR). In addition, reference lists of included studies were checked manually for potential missed publications. We checked whether these studies were included in the original search or not.

Subsequently, data extraction was performed by one reviewer (SS) and completely checked by a second reviewer (RS) using a pre-approved and piloted form. Two authors provided additional data upon request when this was not provided in the article. Data extraction included: first author, year of publication, study design, duration of the study and washout period (if applicable), number of participants, participant characteristics (age, sex, and health status), intervention type and duration, control condition, results of glycemia parameters (mean and standard deviation [SD] not shown), and further comments. PE performed in the morning was considered the control condition and PE performed in the afternoon or evening was considered the intervention in all studies. One trial was stratified by chronotype (e.g. morning bird vs. night owl) and was therefore also analyzed in strata of chronotype. Uncertainties in data extraction were resolved through discussion or the help of a third reviewer (FR).

### Quality assessment

To assess the methodological quality of the included studies, an adapted version of the Quality Assessment Tool for Quantitative Studies was used, as developed by the Effective Public Health Practice Project (EPHPP) [20]. The adaptation of Mackenbach et al. [21] makes the nineteen-item tool suitable for assessing the quality of studies of both observational and experimental designs. It rates the following domains: (1) study design; (2) blinding; (3) representativeness regarding selection bias; (4) representativeness regarding withdrawals/dropouts; (5) confounding; (6) data-collection; (7) data-analysis; and (8) reporting, as ‘weak’, ‘moderate’ or ‘strong’. However, blinding could not be assessed in any of the included studies as it was not possible to blind participants due to the combination of the timing and nature of the intervention. Moreover, blinding of outcome assessors has almost never been mentioned. When included in the rating, most studies would automatically be of moderate to low quality of evidence, although this does not solely determine the quality of the study.

Assessment of selection bias of the participants was based on the representativeness of the participants for a wider population: large study populations with generalizable study characteristics (e.g. >50 participants, large age range, both males and females, etc.) were rated ‘strong’; smaller study populations with less generalizable study characteristics were rated ‘moderate’ (e.g. groups of 10–20 participants, with age ranges 5–10 years); studies with a selected group without generalizable characteristics were rated ‘weak’ (e.g. restricted age groups (< 5-year old ranges), only one gender, only twins). Confounding was assessed as ‘strong’ in randomized designs and cross-over designs, while in non-randomized intervention designs age and sex needed to be adjusted for in order to be rated ‘strong’; adjustment for either was rated ‘moderate’; no adjustment was rated ‘weak’.

Final results from this tool led to an overall ‘weak’, ‘moderate’, or ‘strong’ quality rating based on the assessment of the separate above-mentioned components and the number of ratings given. ‘Strong’ was assigned to those with no ‘weak’ ratings and at least four ‘strong’ ratings; ‘moderate’ to those with one ‘weak’ rating or fewer than four ‘strong’ ratings; and ‘weak’ to those with more than one ‘weak’ rating. Two reviewers (SS and RS) independently assessed the studies for methodological quality, and compared the ratings to reach consensus. Mean proportion of initial agreement between reviewers was 94.8%. Disagreements were discussed until consensus and discrepancies were resolved through discussion with a third reviewer (FR).

### Data synthesis

Studies were meta-analyzed if at least three studies described the same outcome measure. A random-effects model was used due to differences in the methodology of the studies [22]. Due to heterogeneity of the study design, studies comparing one bout of PE (acute studies) were analyzed separately from studies comparing multiple bouts of PE over a period of 5 days to 12 weeks (long-term studies). Furthermore, cross-over studies were only available comparing intervention and control in all participants and were not analyzed based on the specific order in which the condition were administered. Given the limited cross-over effects, including participants in both conditions appears suitable to maintain the sample size in each study [23]. The pooled mean differences were illustrated using forest plots. A p-value of < 0.05 was considered statistically significant.

Statistical heterogeneity was assessed using the I^2^ statistic, where 0% reflects no heterogeneity and 100% reflects complete heterogeneity [22]. A score of more than 75% was considered to indicate substantial heterogeneity. Publication bias was evaluated using Egger’s regression and by visual inspection of funnel plots [22]. The level of evidence was assessed using Grading of Recommendations, Assessment, Development and Evaluation (GRADE) criteria [24]. All studies were trials, so the quality started as high, as is customary with GRADE. The assessment followed the guidelines described in the GRADE handbook and the series of reviews by Guyatt and colleagues [25–32]. In addition, sensitivity analyses were performed to explore the influence of intensity of PE, health status, study quality and chronotype specific PE on the pooled estimates and heterogeneity. No other sources of heterogeneity could be assessed. All analyses and plots were performed using the meta [33] package in R (version 4.3.2) [34]. For a number of studies, pooling was not possible for several reasons, such as missing information in the data extraction or insufficient studies with the same outcome for pooling. These studies were included in qualitative syntheses.

In all analyses, pooled mean differences were calculated by obtaining the difference between the results from the afternoon or evening PE and the morning PE. Beta coefficients were calculated by subtracting the intervention from the control. Therefore, a positive beta reflects a higher glycemia outcome when PE was performed in the morning, whereas a negative beta reflects a higher outcome when PE was performed in the afternoon or evening.

## Results

### Description of included studies

**Fig. 1:**
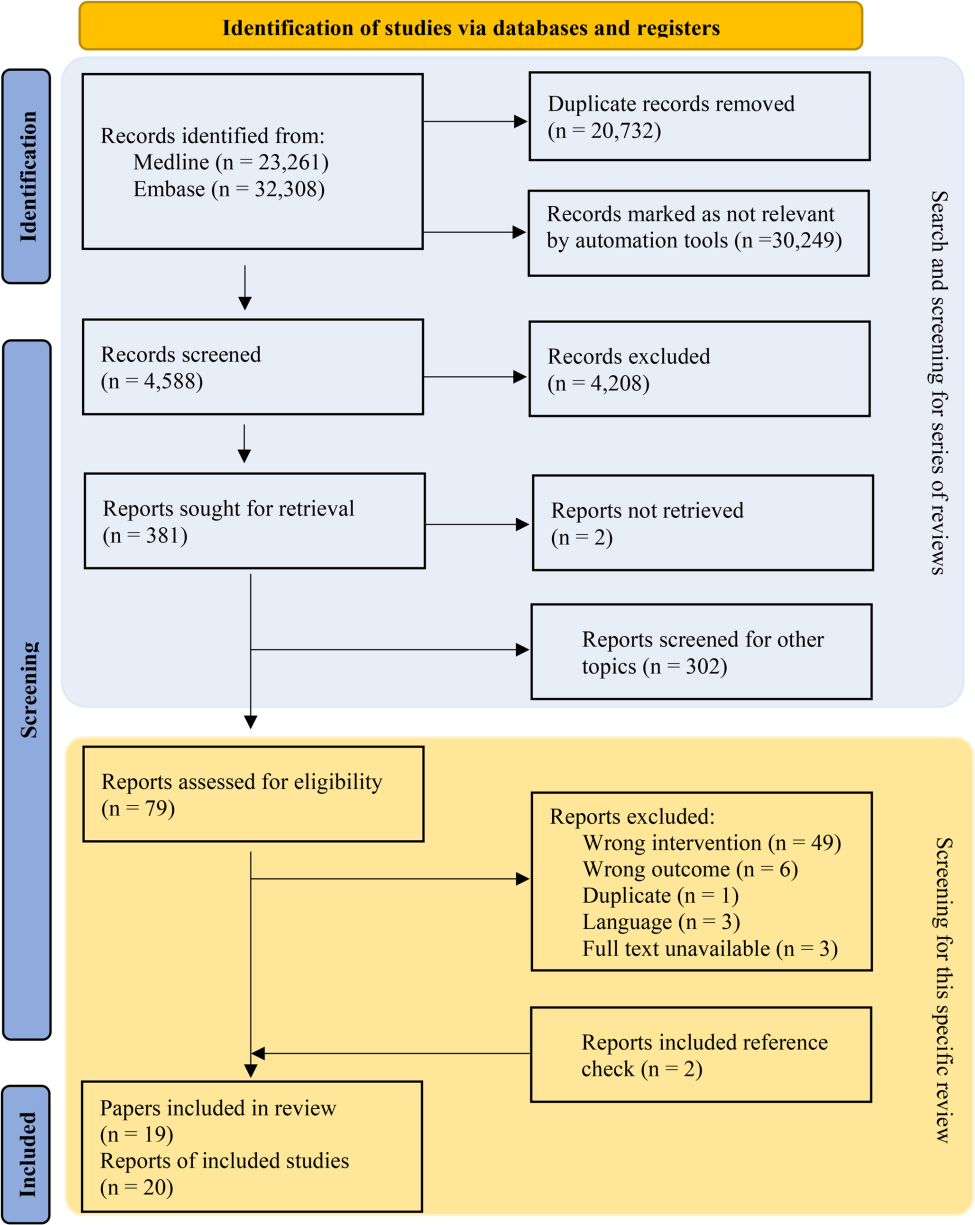
Flow-chart of the search and selection process. Note: This study is part of a series of systematic reviews and meta-analyses. The flow-chart therefore partly consists of data from the series (highlighted in blue). From ‘Reports assessed for eligibility’, only papers for this specific meta-analysis are reported (highlighted in yellow)

A total of 55,569 publications were identified from the systematic literature search. 20,732 duplicate publications and 30,249 studies that were marked as ineligible by the screening tool ASReview were removed [18]. After manually screening 4,588 publications based on title and abstract, 383 full-text publications were read and screened for the topics in our series of systematic reviews and meta-analyses. Of these, 79 articles were potentially relevant for the current review and were read in the full text. Subsequently, 17 publications met the inclusion criteria for this review. Reasons for exclusion after full-text screening are listed in Appendix S2. Two relevant publications were found in the reference lists of included studies, which were not identified in the original search [35, 36]. One papers [37, 38] provided data for two separate studies, making data extraction possible for a total of 20 different studies (Fig. 1).

A total of 680 participants were included from studies in lean participants without a metabolic disorder (*n* = 10 studies), participants with diabetes (*n* = 7 studies), or participants classified as overweight or obese (*n* = 3 studies). Two out of eight studies performed in people with diabetes reported the use of glucose-lowering agents, one study consisting of two subgroups did not specify its use [38]. The age of participants ranged from 21 to 66 years and 54.1% were men. In one study, the sex distribution was not reported [39]. Different types of PE were assessed, namely walking (*n* = 2 studies), cycling (*n* = 9 studies), resistance exercise (RE) (*n* = 1 study), running (*n* = 5 studies), a combination of walking or cycling and RE (*n* = 2 studies), or yoga (*n* = 1 studies). Twelve studies, referred to as acute studies, examined the acute effects of timing of PE either because each experimental condition was studied for one day in a randomized crossover design (*n* = 11 studies) or in a non-randomized crossover design (*n* = 1). Eight studies, referred to as long-term studies, examined the effect of timing of PE over multiple bouts of exercise in a period ranging from 5 days to 12 weeks, where participants were randomized (*n* = 5 studies) or non-randomized (*n* = 3 studies) into a morning or afternoon/evening PE. Duration of PE intervention ranged from approximately 2 min to 120 min per PE bout, varying from high intensity aerobic exercise (e.g. 1000 m sprint) to resistance-type exercise and walking or yoga. A detailed overview of the study characteristics is provided in Table 1. Finally, methodological quality was considered strong in nine studies, moderate in ten studies, and weak in one study (Table 2). The most important reason for risk of bias included lack of information about withdrawals and dropouts.

**Table 1:** Study characteristics (AUC: area under the curve; CGM: continuous glucose monitoring; E: early physical exercise; EC: evening chronotype F: Female; HOMA-IR: Homeostatic Model Assessment for Insulin Resistance; L: late physical exercise; M: male; MAGE: Mean amplitude of glycemic excursions; ME: morning chronotype; N: study population; NR: not reported; SD: Standard Deviation; T1D: Type 1 Diabetes; T2D: Type 2 Diabetes; YYIRT: Yo-Yo Intermittent Recovery Test)

Author and year	Study design	Study polulation	*N*	Sex (M/F)	Age	Follow-up time	Intervention	Control	Glycemic outcomes	Comments
Acute studies										
Aloui et al., 2017 [39]	Randomized cross-over	Healthy	11	NR	21.00 ± 0.48	One exercise per trial; ≥36 h recovery period	YYIRT at 17.00 h.	YYIRT at 7.00 h.	Blood samples (glucose) pre-exercise and 3 min post-exercise	Fasting time NR. Meals replicated in trials
Fernandes et al., 2014 [40]	Randomized cross-over	Healthy, recreational cyclists	9	(9/0)	31 ± 7.3	One exercise per trial; 7 days recovery period	1000 m cycling time trial at 18.00 h after 6 h fast	1000 m cycling time trial at 8.00 h after 8 h fast	Blood samples (glucose, glucagon and insulin) pre-exercise, immediately post and 60 min post-exercise	Intake and its timing replicated 48 h prior to trial
Galliven et al., 1997.1 [37]	Randomized cross-over	Healthy	9	(0/9)	29 ± 3	One exercise per trial; ≥7days recovery period	20 min high-intensity treadmill exercise between 15.00–16.00 h after 6 h fast	20 min high-intensity treadmill exercise between 7.00–8.00 h after overnight fast	Blood samples (glucose) 20 and 10 min pre-exercise, midway exercise, immediately, 10, 20, 40 and 60 min post-exercise. AUC glucose	
Galliven et al., 1997.2 [37]	Randomized cross-over	Healthy	8	(0/8)	31 ± 2,83	Two menstrual cycles, 1 bout of PE per menstrual phase (day 3–9, 10–16, 18–26)	20 min moderate-intensity treadmill exercise between 15.00–16.00 h after 6 h fast	20 min moderate-intensity treadmill exercise between 7.00–8.00 h after overnight fast	Blood samples (glucose) 20 and 10 min pre-exercise, midway exercise, immediately, 10, 20, 40 and 60 min post-exercise. AUC glucose	
Gomez et al., 2015 [41]	Randomized cross-over	T1D	32	(17/15)	30.31 ± 12.66	One exercise per trial; 7–14 days recovery period	4 × 15 min moderate treadmill exercise at 16.00 h after 4 h fast	4 × 15 min moderate treadmill exercise at 7.00 h after overnight fast	CGM data (Time in euglycemia, hypo- and hyperglycemic events) up to 36 h post-exercise	Standardized intake prior to trial and one meal after trial
Hobson et al., 2009 [47]	Randomized cross-over	Healthy, recreationally active	9	(9/0)	24 ± 2	One exercise per trial; 7 days recovery period	cycling to exhaustion at 65% V˙ O2peak in an ambient temperature of 35-C (60% relative humidity) at 18.45 h after 6 h fast	cycling to exhaustion at 65% VO2peak in an ambient temperature of 35-C (60% relative humidity) at 06.45 h after 6-10 h fast	Blood samples (fasting glucose) pre-exercise and every 15 min thereafter during exercise.	Standardized intake 48 h prior to trial
Larsen et al., 2019 [35]	Randomized cross-over	Overweight and inactive	11	(11/0)	49 ± 5	One exercise per trial; ≥5 days recovery period	30 min of HHIE on cycle ergometer between 14.00–16.00 h after 3 h fast	30 min of HHIE on cycle ergometer between 6.00–7.00 h after overnight fast	Capillary glucose prior, 30 post-exercise, fasting blood sample (glucose) day after.	Evening trial was not included in analyses
Li & Gleeson, 2004 [43]	Non-randomized cross-over	Healthy, recreationally active	8	(8/0)	28.9 ± 5,09	One exercise per trial ≥ 4 days in between	cycling for 2 h at 60% VO2max at 14.00 h after 15 h fast	cycling for 2 h at 60% VO2max at 9.00 h after 10 h fast	Fasting blood samples (glucose) 5 min prior to and immediately post-exercise	Second exercise during the day at noon and control trial were not included in analyses
McIver et al., 2021 [46]	Randomized cross-over	Healthy, recreationally active	12	(12/0)	25 ± 3	One exercise per trial; ≥7days recovery period	45 min brisk walking on level motorized treadmill at 15.00 h after 8 h fast	45 min brisk walking on level motorized treadmill at 8.00 h after 8 h fast	Capillary blood samples (glucose, glucose iAUC) baseline, post-breakfast, prior to and immediately after exercise, pre-lunch ingestion, then every 30 min post-lunch ingestion	Only fasted trials were included in analyses
Munan et al., 2020 [44]	Randomized cross-over	T2D	14	(8/6)	65 ± 9	In 12 days	50 min walking at 5.0 km/h 3–4 h after lunch ending 20 min before dinner	50 min walking at 5.0 km/h ending 20 min before breakfast	CGM (24 h glucose, fasting glucose, 2 h post-prandial glucose, 24 h MAGE)	Control trial not included in analyses Standardized meal on day of trial and one day thereafter
Ruegemer et al., 1990 [42]	Randomized cross-over	T1D	6	(3/3)	30 ± 9.8	One exercise per trial; in 3–4 months period	30 min cycle ergometer exercise at ~ 60% of Vo2max at 16.00 h after 4 h fast	30 min cycle ergometer exercise at ~ 60% of Vo2max at 7.00 h after overnight fast	Blood samples (glucose, glucagon, insulin) at -20, -10, 0, 15, 30, 45, 60, 75, 90, 120, 180, 240, 300, and 360 min, AUC glucose, AUC insulin	Standardized meal on day of trial
Tanaka et al., 2021 [45]	Randomized cross-over	Healthy	11	(11/0)	24.5 ± 2.8	One exercise per trial; ≥ 7 days recovery period	1 h ergometer cycling at 60% VO2max at 16.00 h, 3 h after lunch	1 h ergometer cycling at 60% VO2max at 7.00 h before breakfast	CGM (24 h SD, J-index, MAGE)	Control trial not included in analyses. Standardized meal on day of trial
Long-term studies										
Brooker et al., 2023 [36]	RCT	Overweight/obese and inactive	Total: 80; E:40 ; L:40	(21/59)	E: 41 ± 12; L: 38 ± 11	12 weeks	250 min/week of self-paced aerobic (treadmill-based) exercise between 16.00–19.00 h	250 min/week of self-paced aerobic (treadmill-based) exercise between 6.00–9.00 h	Capillary blood samples (fasting glucose) at baseline and 12 weeks	Control trial not included in analyses
Kim et al., 2022 [53]	Randomized cross-over	Healthy	12	(12/0)	21.8 ± 0.7	3 exercises per trial in one week; 2 weeks recovery period	60 min supervised treadmill exercise at 60% VO2 max at 16.00–18.00 h	60 min supervised treadmill exercise at 60% VO2 max at 9.00–11.00 h	CGM (24 h mean glucose, min glucose, max glucose, J-index, MAGE, AUC 4 h postprandial), blood samples (fasting glucose and insulin pre-intervention and > 48 h post intervention)	
Krcmárová et al., 2018 [48]	Parallel group randomized controlled trial	Healthy	Total: 20; E:10 ; L:10	(0/20)	66 ± 4	12 weeks, 2x/week	Whole body strength training: 3 sets of 10–12 repetitions with 2–3 min rest between sets at 18.00 h	Whole body strength training: 3 sets of 10–12 repetitions with 2–3 min rest between sets at 7.30 h	Blood samples (glucose) after 12 h fasting 7 days prior to and 7 days after training period	Control trial not included in analyses
Mancilla et al., 2020 [49]	Retrospective trial	At risk for or diagnosed with T2D	Total: 32, E: 12, L: 20	(32/0)	total:58 ± 7, E: 61 ± 5, L: 57 ± 7	12 weeks, 3x/week	2x/week 30 min aerobic ergometer cycling for 30 min at 70% workload and 1x/week 3 series of 10 repetitions at 60% maximal voluntary contraction between 15.00–18.00 h	2x/week 30 min aerobic ergometer cycling for 30 min at 70% workload and 1x/week 3 series of 10 repetitions at 60% maximal voluntary contraction between 8.00–10.00 h	Blood samples (glucose), hyperinsulinemic-euglycemic clamp 48 h pre- and 48–72 h post-exercise, peripheral insulin sensitivity	
Menek et al., 2022 [38]	Non-randomized cross-over	T2D	Total: 30 MC: 15. EC: 15	(11/19)	Range 35–65	3x/week over 6 weeks; no wash-out period	structured aerobic and resistance exercise program 1 h after dinner	structured aerobic and resistance exercise program 1 h after breakfast	Blood samples (hba1c, fasting blood glucose) at baseline and after 6 weeks	Analyses stratified by chronotype
Moholdt et al., 2021 [50]	Parallel group randomized controlled trial	Overweight/obese	Total: 16; E: 8 L: 8	(16/0)	E: 35 ± 4, L: 36 ± 5	Once-daily exercise for 5 consecutive days after 5 days of HFD	day 1,3,5: high-intensity cycling 10 × 1 min; day 2,4: moderate-intensity cycling 40 and 60 min, respectively, at 18.30 h before dinner	day 1,3,5: high-intensity cycling 10 × 1 min; day 2,4: moderate-intensity cycling 40 and 60 min, respectively, at 6.30 h before breakfast	CGM (24 h glucose), blood samples (fasting glucose, insulin) at baseline and post 5 days exercise, calculated HOMA-IR	Control trial not included in analyses
Teo et al., 2020 [51]	Parallel group randomized controlled trial	Sedentary and overweight with and without T2D	T2D:20; no T2D: 20	(17/23)	51 ± 13	3x/week; 12 weeks	supervised 60 min exercise training (30 min treadmill walking and 30 min resistance exercise) sessions between 17.00–19.00 h	Supervised 60 min exercise training sessions between 8.00–10.00 h	Fasting blood samples (glucose, HbA1c, insulin, HOMA2-IR, postprandial glucose AUC, postprandial insulin AUC) before and after exercise period and every 15 min for 2 h after test meal	
Vijayakumar et al., 2018 [52]	Non-randomized trial	With and without T2D	T2D: 189; no T2D: 121	T2D (112/77); no T2D (59/62)	T2D male: 54.31 ± 11.28; T2D female: 54.91 ± 10.67; no T2D male: 51.37 ± 8.45; no T2D female: 47.35 ± 8.54	One session daily, 10 days	daily yoga program (60 min practical and 30 min theoretical session) at 17.30 h	Daily yoga program (60 min practical and 30 min theoretical session) at 5.30 h	Blood samples (fasting glucose) before day 1 and after day 10	

**Table 2:** Overview quality assessment (NR: Not reported)

Author	Design	Blinding	Selection bias	Withdrawals and dropouts	Confounders	Data collection	Data analysis	Reporting	Overall
Aloui et al. [39]	Strong	NR	Moderate	Weak	Strong	Strong	Strong	Moderate	**Moderate**
Fernandes et al. [40]	Strong	NR	Moderate	Weak	Strong	Strong	Strong	Moderate	**Moderate**
Galliven et al. 1 [37]	Strong	NR	Moderate	Weak	Strong	Strong	Strong	Moderate	**Moderate**
Galliven et al. 2 [37]	Strong	NR	Moderate	Weak	Strong	Strong	Strong	Moderate	**Moderate**
Gomez et al. [41]	Strong	NR	Moderate	Strong	Strong	Strong	Strong	Strong	**Strong**
Ruegemer et al. [42]	Strong	NR	Weak	Weak	Strong	Strong	Strong	Moderate	**Weak**
Krcmárová et al. [48]	Strong	NR	Moderate	Strong	Strong	Strong	Strong	Moderate	**Strong**
Li&Gleeson [43]	Strong	NR	Moderate	Weak	Strong	Strong	Strong	Strong	**Moderate**
Mancilla et al. [49]	Strong	NR	Moderate	NR	Weak	Strong	Strong	Strong	**Moderate**
McIver et al. [46]	Strong	NR	Moderate	Weak	Strong	Strong	Strong	Strong	**Moderate**
Moholdt et al. [50]	Strong	NR	Moderate	Moderate	Strong	Strong	Strong	Strong	**Strong**
Munan et al. [44]	Strong	NR	Moderate	Moderate	Strong	Strong	Strong	Strong	**Strong**
Tanaka et al. [45]	Strong	NR	Moderate	Weak	Strong	Strong	Strong	Strong	**Moderate**
Teo et al. [51]	Strong	NR	Moderate	Strong	Strong	Strong	Strong	Strong	**Strong**
Vijayakumar et al. [52]	Strong	NR	Moderate	Weak	Moderate	Strong	Strong	Strong	**Moderate**
Hobson et al. [47]	Strong	NR	Moderate	Strong	Strong	Strong	Strong	Strong	**Strong**
Kim et al. [53]	Strong	NR	Moderate	Moderate	Strong	Strong	Strong	Moderate	**Strong**
Menek et al. [38]	Strong	NR	Strong	Strong	Weak	Strong	Strong	Strong	**Moderate**
Larsen et al. [35]	Strong	NR	Moderate	Strong	Strong	Strong	Strong	Strong	**Strong**
Brooker et al. [36]	Strong	NR	Strong	Strong	Strong	Strong	Strong	Strong	**Strong**

### Acute studies

**Fig. 2:**
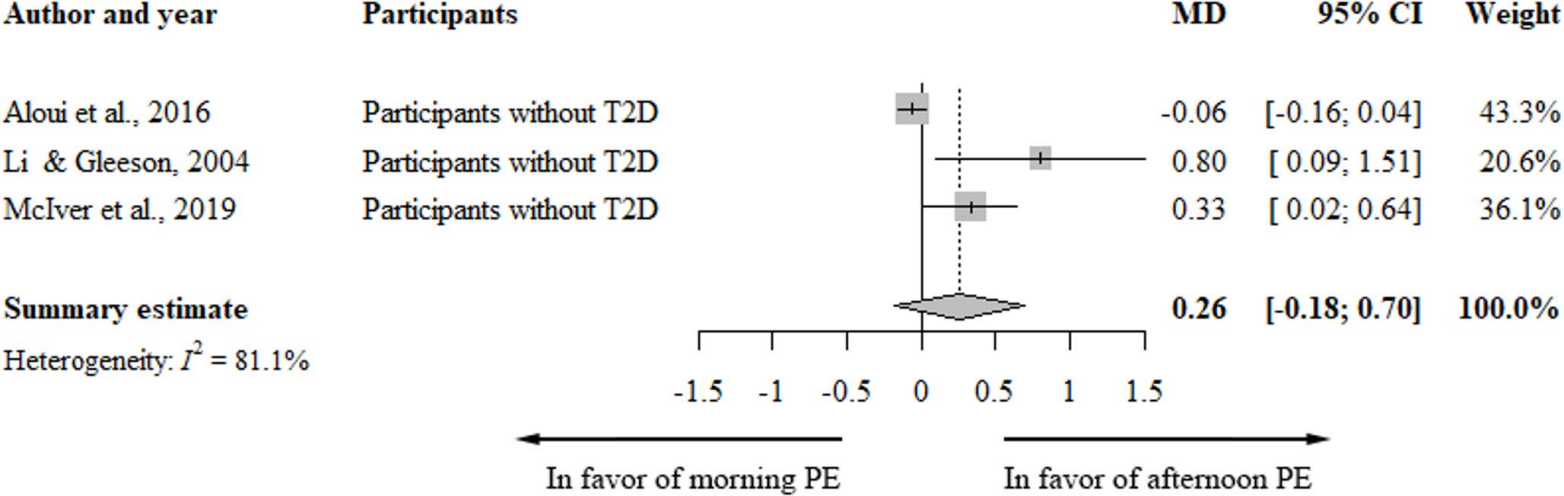
Forest plot blood glucose directly after physical exercise in acute studies. Data presented as pooled mean difference (MD; mmol/L); CI: Confidence Interval; PE: Physical Exercise; T2D: Type 2 Diabetes

Of the twelve acute studies [35, 37, 39–47], three studies that reported on glucose concentrations immediately after PE were meta-analyzed [39, 43, 46]. Figure 2 shows a slightly, but non-significantly higher blood glucose levels after PE in those undertaking morning, compared to afternoon PE (0.26 mmol/L, [-0.18; 0.70], I^2^ = 81%). Two studies that reported on glucose levels immediately after PE could not be included in the meta-analysis, but qualitative evaluation showed similar statistically significant effects [40, 47]. Sensitivity analyses could not be performed because only three studies were included in the analysis. The level of evidence as measured by GRADE [25] was of low quality because of inconsistency as a result of variability in results with point estimates widely varying across studies, and indirectness based on variation in intervention.

Other outcomes from acute studies could not be meta-analyzed due to less than three studies reporting on the same outcome. Qualitative systematic review of these studies showed no difference in 24-hour mean glucose [44] and either no differences [40] or higher glucose 30–60 min after morning versus evening PE [35, 42]. When expressed as glucose AUC over a period of 60–150 min, incremental glucose AUC or related glucose outcomes (i.e. peak increment), findings were inconsistent. No differences were observed in two studies [37, 46] and one study reported higher increments after morning versus afternoon PE [42].

Regarding additional glucose measures, two studies reported no significant differences in either 24-hour standard deviation of glucose, coefficient of variation, J-index, continuous overall net glycemic action (CONGA), Homeostatic Model Assessment for Insulin Resistance (HOMA-IR), correlation property of glucose fluctuation (DFa) or mean amplitude of glycemic excursions (MAGE) between morning and afternoon or evening PE [44, 45]. Two studies assessed glucagon and insulin concentrations at multiple time points, generally showing no consistent differences [40, 42]. Only Ruegemer et al. reported a statistically significant decrease in insulin levels that were measured every 10 min during afternoon PE, which was not found during morning PE [42]. When expressed as insulin area under the curve, insulin levels were significantly higher during afternoon or evening PE than during morning PE, suggesting more pancreatic insulin secretion [42].

Furthermore, one study reported an improvement in time in range (TIR), defined as glucose levels between 3.9 and 10.0 mmol/l, the day after morning PE compared to the day prior to PE based on CGM data measured over the 36 h after exercise, which was not found after afternoon PE [41]. Secondly, one study investigated the time spent in hyperglycemia based on 24 h CGM data and found no significant differences [44]. With regard to time spent in hypoglycemia, Gomez et al. observed more time spent in hypoglycemia the day after afternoon PE compared to morning PE in people with type 1 diabetes [41].

### Long-term studies

**Fig. 3:**
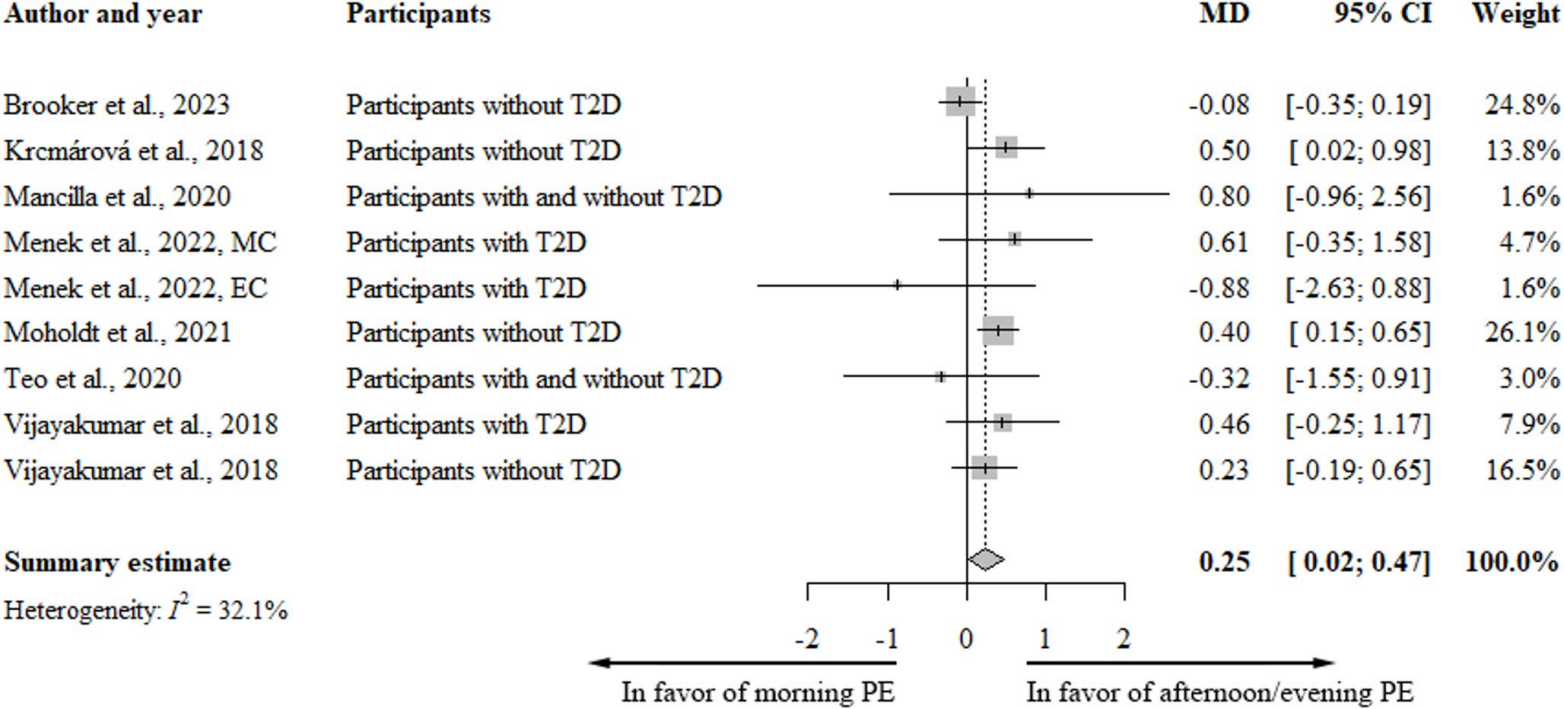
Forest plot fasting blood glucose in long-term studies. Data presented as pooled mean difference (mmol/L); CI: Confidence Interval; EC: evening chronotype; MC: morning chronotype; PE: Physical Exercise; T2D: type 2 diabetes

Eight long-term studies investigated the effect of differences in timing of PE on fasting glucose concentration over multiple bouts of exercise over a period of 5 days to 12 weeks [36, 38, 48–53], which could all be meta-analyzed as presented in Fig. 3. The meta-analysis showed a statistically significant difference in overnight fasting glucose (measured after the intervention period) of 0.25 mmol/L ([ 0.02–0.47], I^2^ = 32%) higher concentrations after morning versus afternoon or evening PE. Sensitivity analyses on health status, exercise intensity, and study quality did not reduce heterogeneity or change the estimates, neither did excluding chronotype specified exercise (Appendix S3) [38]. The level of evidence as measured by GRADE [25] was of moderate quality, because of indirectness based on variation in intervention.

Other outcomes were analyzed qualitatively due to fewer than three studies reporting on the same outcome, or in the case of HbA1c, were measured over a time period that was too short to be a reliable outcome and thus not reported here [38]. One study reported increased glucose concentrations across multiple time points, when morning PE was compared to evening PE [50]. When expressed as 4 h glucose AUC after PE, one study found significantly lower glucose AUC after afternoon or evening PE, compared to morning PE [51], while another study found no differences [53]. Furthermore, two studies reporting on HbA1c levels found no differences when morning PE was compared to evening PE [38, 51].

For other glycemic measures, two studies assessing HOMA-IR found no differences [10, 50], while one study reported a statistically significant lower J-index after afternoon or evening PE but no significant differences in MAGE [53]. One study reported statistically significant improved peripheral insulin sensitivity and greater insulin-mediated suppression of adipose tissue lipolysis following afternoon or evening PE compared to morning PE, although effects on hepatic glucose output were not statistically significant (*p* = 0.057) [49].

Finally, two studies assessing insulin concentrations (fasted or 4 h AUC) reported no significant differences between timing conditions [50, 51].

### Publication bias

As we only included a few studies per outcome, funnel plots were not useful to assess publication bias (Appendix S4). Egger’s test confirmed the absence of asymmetry in fasting glucose of long-term studies (*p* = 0.9418), and in glucose directly after PE from acute studies (*p* = 0.0779). However, it should be noted that both funnel plots and Egger’s test is discouraged due to limited power when fewer than ten studies are included [22, 23, 54]. So these results should be interpreted cautiously.

## Discussion

We systematically reviewed and meta-analyzed 20 studies to investigate the effect of time of day of PE on glycemia parameters. The results from acute studies were highly variable, with wide confidence intervals and substantial heterogeneity, rendering the findings inconclusive. Although some data suggested a tendency toward higher glucose directly after morning PE, compared with afternoon or evening PE, the evidence for acute effects remains tentative. In contrast, long-term studies showed a statistically significant higher fasting glucose following morning PE, compared to afternoon or evening PE, suggesting potentially less favorable effects on glucose metabolism after (prolonged) morning PE. Qualitative synthesis of indices of blood glucose homeostasis were inconsistent or supported the meta-analysis, except for TIR and hypoglycemic events in acute studies, which appeared to be more in favor of morning PE. The latter might possibly be explained as a result of a smaller glucose lowering effect of PE when performed in the morning. Our results regarding glucose are in line with previously conducted reviews [11, 12], which have shown similar effects on cardiovascular health and blood glucose. However, we extended with data on other glycemia parameters.

The high heterogeneity in our results is in part explained by the broad definitions we used in our search to provide a comprehensive overview of the available literature, leading to large variability in study design. We would like to emphasize that this review is intended to generate an overall impression of the order and magnitude of the association, rather than a precise estimate. Furthermore, we included one non-randomized study that was stratified by chronotype without a washout period that showed most beneficial effects of PE at the chronotype appropriate timing [38]. However, the chronotype appropriate intervention was the first intervention performed for both arms, after which blood glucose continued to decrease. So results may be biased by the order of intervention which make it seem that the chronotype inappropriate timing is most beneficial for lowering glucose levels. Nevertheless, sensitivity analysis excluding this study did not alter the results. Additionally, some differences with previous reviews should be highlighted. First, we included studies compromised of participants with and without T2D. Research has shown that people with T2D have altered diurnal rhythms of glucose tolerance, compared to individuals without diabetes [55, 56], which may lead to differences in results between people with and without T2D. However, sensitivity analysis on health status did not reduce heterogeneity or affect the estimates. Second, although within studies, the type of intervention was the same, some studies found differences in physical performance between morning and afternoon PE (e.g [39, 40, 48]). Physical performance is shown to be time-dependent, with enhanced performance in the afternoon, which coincides with a higher metabolic rate. The underlying mechanism of enhanced performance in the afternoon is not completely understood, but a slightly increased core temperature in the evening could increase nerve conduction velocity and vasodilatation, which in turn increases muscular supply and substrate elimination, thereby improving glycogenolysis and glycolysis, lowering plasma glucose. (e.g [40, 57]). Third, included studies did not all standardize food intake and/or fasting periods before PE (despite excluding studies with energy intake < 3 h before PE, with one study not reporting on fasting time at all [39]). For instance, McIver et al. is the only acute study that asked participants to replicate their food intake the day prior to the trials in combination with a fasting period of 8 h before each trial [46]. Therefore, PE after three- or four-hour fasting was compared to PE after overnight fasting in some acute studies. Consequently, the effect of time of day may in part be altered by the effect of energy-intake, for example through increased hepatic glucose release upon exercise in the fasted state, but not in the fed state [49]; more free fatty acid utilization through increased levels of adrenaline, noradrenaline and growth hormone in fasted PE compared to fed PE; or the activation of metabolic signaling pathways beneficial for metabolic adaptions in skeletal muscles in fast PE. These potential mechanisms caused by heterogeneity between the studies should be taken into account when interpreting the results.

With regard to mechanisms explaining these differences in timing of PE on glycemia parameters, there are several hypotheses. First, several studies suggest that exercise enhances glucose uptake and utilization through mechanisms that may be enhanced in the dark phase (i.e. late hours). This includes IL-6 induced glucose uptake through glucose transporter GLUT4, which causes peak muscular strength and muscle mitochondrial function in the afternoon [12, 49, 53, 58, 59]. Glucose utilization may be enhanced in the afternoon as a result of higher insulin-mediated suppression of adipose tissue lipolysis, which causes less free fatty acids to be available for oxidation, thereby enhancing glucose oxidation [49, 58]. Moreover, uncontrolled adipose tissue lipolysis is associated with the development of skeletal muscle insulin resistance [60]. However, it must be noted that previous studies have indicated that the effects of PE on glucose levels were consistent with differences observed in basal endogenous glucose production upon exercise timing, where basal endogenous glucose production was profoundly affected by the distribution and composition of pre-exercise energy intake. So that may be a confounding factor that we could not account for in the present study but that could alter results significantly. Second, cortisol and free insulin levels differ throughout the day, with higher levels in the morning [12]. Even though insulin is expected to have a counter reactive effect on cortisol, it is suggested that cortisol is negatively associated with compensatory mechanisms for insulin resistance such as β-cell function and insulin release thereby elevating blood glucose levels [61]. Morning PE may coincide with the morning cortisol peak, which may explain the less favorable glycemia parameters when compared to afternoon or evening PA. Furthermore, the lower cortisol concentration in the afternoon and particularly at midnight may explain the increased incidence of hypoglycemic events after late PE through reduced gluconeogenesis and reduced glucagon response as a result of relative higher insulin sensitivity [41].

### Strengths and limitations

Our systematic review and meta-analysis provides an extensive overview of the effect of the timing of PE on glycemia parameters using multiple markers. To our knowledge, this is the first systematic review that compares multiple glycemia parameters, providing a broader overview of the available evidence on the effect of the timing of PE on glycemia parameters. Furthermore, this systematic review and meta-analysis was conducted in accordance with the PRISMA guidelines.

Nevertheless, several limitations should be noted. First, in the acute studies, we could only meta-analyze glucose directly after PE, as too few studies reported on other glycemia parameters. Even for glucose, not all studies could be included because necessary data were lacking, which may limit the representativeness and the robustness of the findings. Second, despite using an extensive a priori search strategy, two papers were retrieved from reference lists rather than the original search. This may be due to missing phrase indexation and keywords, which may have limited their retrieval through the search strategy. Third, crossover studies were included and may be subject to carry-over effects, but adequate wash-out periods were described in most studies [23]. Therefore, it is unlikely to have substantially influenced the outcomes. Fourth, different methods of measuring blood glucose were used (e.g. continuous glucose monitor (CGM) or intravenous (iv) catheters). Studies have shown a physiological time delay of 5–8 min between blood glucose from iv catheter and interstitial glucose from CGMs, resulting in slightly different absolute concentrations between studies, but this is unlikely to affect within-study comparisons [62, 63]. However, some studies have also criticized the use of CGMs reporting that the placement of the CGM significantly changed mean glucose levels [64, 65] and measures of glycemic variability derived from CGM may differ from venous blood data [62]. Although differences may be small, future studies should take the validity of the glucose measurements as well as time delays into account.

### Clinical implications

Current Diabetes Guidelines [66–68] do not include recommendations on the timing of PE. Our findings suggest that afternoon or evening PE may be more beneficial than morning PE with regard to glycemia parameters. However, the observed mean difference of 0.25 mmol/L in fasting glucose, although statistically significant, is small and of uncertain clinical relevance, and the evidence is derived from a limited number of small and heterogeneous studies. While several individual studies suggest a trend toward lower glucose after evening PE, the consistency and magnitude of this effect remains uncertain, highlighting the need for larger, well-controlled trials to clarify the true clinical impact of exercise timing on glycemic regulation. Therefore, RCTs with larger groups (e.g. >50 participants) and longer follow-up periods (e.g. >6 months) using more clinically relevant outcomes such as HbA1c, are needed to further investigate the long-term effects of the timing of PE on glycemia parameters and the sustainability of these effects.

As mentioned before, factors such as endogenous glucose production and nutritional intake may partially explain the difference in results and represent important limitations in the existing literature. Therefore, these factors should be specifically addressed in further research. Furthermore, several authors have reported that sleep quality and duration may influence insulin resistance through hormonal, behavioral and circadian mechanisms, but none of the included studies have investigated sleep while exercise, especially in the evening, may affect sleep. Additionally, only few studies have taken chronotype into account, while the optimal timing may differ per chronotype [38–40]. Therefore, other lifestyle factors besides nutrition (e.g. sleep, chronotype) should also be considered as these may influence circadian patterns and thus the effect of PE at a given timepoint [4].

Moreover, this systematic review and meta-analysis only focused on glycemia parameters and not on lipid metabolism outcomes, which may influence glucose metabolism and potentially contribute to observed glycemic responses. This highlights an important gap in the literature that needs addressing.

## Conclusion

This review suggests that fasting glucose may be higher following morning PE compared with afternoon or evening PE in long-term studies. Evidence on glucose directly after PE in acute studies and other glycemic outcomes remains limited and inconclusive. The included studies were generally small, heterogeneous in design and interventions, and often did not account for important factors such as nutritional intake and chronotype. Therefore, well-designed, adequately powered trials that account for lifestyle factors and assess clinically relevant outcomes are needed to clarify these associations.

## Electronic Supplementary Material

Below is the link to the electronic supplementary material.

Supplementary Material 1

## Data Availability

The data, code and other materials that underlie the results reported in this article are available from hoornstudy@amsterdamumc.nl reasonable request to researchers who provide a methodological sound proposal and after approval by the Hoorn Steering Committee.

## Abbreviations

ADA: American Diabetes Association
AUC: area under the curve
CGM: continuous glucose monitor
CONGA: continuous overall net glycemic action
DFa: correlation property of glucose fluctuation
EPHPP: Effective Public Health Practice Project
GRADE: Grading of Recommendations, Assessment, Development and Evaluation
HOMA-IR: Homeostatic Model Assessment for Insulin Resistance
Iv: intravenous
MAGE: Mean amplitude of glycemic excursions
PE: physical exercise
PRISMA: Preferred Reporting Items for Systematic Reviews and Meta-Analyses
RE: resistance exercise
T2D: type 2 diabetes
WHO: World Health Organization
