# Supplementary material for: The effect of timing of physical exercise on glycemia: a systematic review and meta-analysis of human intervention studies
Source: J Diabetes Metab Disord. 2026 May 29;25(1):150. doi: 10.1007/s40200-026-01954-z (PMC13221532; doi:10.1007/s40200-026-01954-z)
Supplement: Supplementary file 1 — Supplementary Material 1 [file 40200_2026_1954_MOESM1_ESM.docx]

S1: Search strategy february 24, 2023

| Database | Results 23 sept 2021 | Results 24 feb 2023 |
| --- | --- | --- |
| Medline (Ovid) | 16,994 | 23,261 |
| Embase.com | 23,677 | 32,308 |
| Total | 40671 | 55569 |
| After deduplication | 27848 | 39411 |

###

### Medline (Ovid) Search History February 24, 2023

| **Set** | **Query Medline (Ovid) – February 24, 2023** | **Results** |
| --- | --- | --- |
| #1 | Diabetes Mellitus/ or exp Diabetes Mellitus, Type 2/ or exp Prediabetic State/ or exp Insulin Resistance/ or exp Hyperinsulinism/ or exp Glucose Intolerance/ or exp Blood Glucose/ or exp Hyperglycemia/ or exp Glycated Hemoglobin A/ or (diabet* or type-2-dm or type-II-dm OR dm-type-2 OR dm-type-II OR NIDDM OR IDDM OR (insulin adj3 (resistan* or sensitiv* or insensitiv*)) or fasting-insulin OR homa-ir OR homa2 OR hyperinsulin* OR prediabet* OR (glucose ADJ3 (intoleran* or toleran* or dysregulat*)) OR igt OR (glucose ADJ3 (blood or plasma or level* or fasting OR postprandial OR post-prandial)) OR "postprandial metabolism*" OR "post-prandial metabolism" OR blood-sugar OR hyperglycem* OR hyperglycaem* OR Hb-A1c OR HbA1c OR Hemoglobin A1c OR glycemi* OR glycaemi* OR area-under-the-curve OR AUC OR HOMA-IR OR QUICKI OR matsuda).ti,ab,kf | 1,153,511 |
| #2 | exp Diet/ or exp Diet Therapy/ or Nutrition Therapy/ or exp Diet, Reducing/ or exp Caloric Restriction/ or exp Feeding Behavior/ or exp Fasting/ or Exercise/ or exp Exercise Therapy/ or exp Sleep/ or exp Sleep Deprivation/ or exp Sleep Wake Disorders/ or exp Sleep Disorders, Circadian Rhythm/ | 786,540 |
| #3 | exp Time/ or exp Time Factors/ or exp Circadian Rhythm/ or exp Chronobiology Disorders/ or (timing OR schedul* OR time* OR morning OR afternoon OR evening OR night OR specific-time OR duration or exten* or intermittent or interval or periodic or alternate-day or (chrono* ADJ3 nutrition) or chrononutrition).ti,ab,kf | 7,947,296 |
| #4 | ((diet or nutrition* or eat* or food* or feed* or fasting OR energy-restriction or caloric-restriction or meal*or chrononutrition or chrono-nutrition or food-intake or big-breakfast OR exercis* OR (physic* ADJ2 activ*) or workout or train* or run* or swim* or walk* or sleep) adj10 (timing OR schedul* OR time* OR morning OR afternoon OR evening OR night OR daytime* OR nighttime* OR specific-time OR duration or shift* or exten* or intermittent or interval or periodic or alternate-day)).ti,ab,kf | 349,617 |
| #5 | Exp Clinical Trial/ OR Exp Health Promotion/ OR Exp Comparative Study/ OR (trial* OR rct OR controlled OR intervention* OR (compar* ADJ3 group*) OR "comparative study" OR "laboratory protocol" OR random* OR crossover OR cross-over OR program* OR support OR advice OR counseling OR counselling OR training OR weight-management OR coaching OR motivational-interview* OR campaign*).ti,ab,kf | 7,622,439 |
| #6 | 2 and 3 | 275,045 |
| #7 | 6 or 4 | 511,704 |
| #8 | 1 and 7 and 5 | 24,694 |
| #9 | 8 not ((Adolescent/ or Child/ or Infant/ or adolescen*.ti,ab,kf. or child*.ti,ab,kf. or schoolchild*.ti,ab,kf. or infant*.ti,ab,kf. or girl*.ti,ab,kf. or boy*.ti,ab,kf. or teen.ti,ab,kf. or teens.ti,ab,kf. or teenager*.ti,ab,kf. or youth*.ti,ab,kf. or pediatr*.ti,ab,kf. or paediatr*.ti,ab,kf. or puber*.ti,ab,kf.) not (Adult/ or adult*.ti,ab,kf. or man.ti,ab,kf. or men.ti,ab,kf. or woman.ti,ab,kf. or women.ti,ab,kf.)) | 23,261 |

### Embase Search History February 24, 2023

| Set | **Query Embase.com – February 24, 2023** | Results |
| --- | --- | --- |
| #1 | 'diabetes mellitus'/de OR 'insulin dependent diabetes mellitus'/exp OR 'non insulin dependent diabetes mellitus'/exp OR 'glucose intolerance'/exp OR 'glucose blood level'/exp OR 'hyperglycemia'/exp OR 'glycosylated hemoglobin'/exp OR 'impaired glucose tolerance'/exp OR 'insulin resistance'/exp OR 'hyperinsulinism'/exp OR (diabet* or type-2-dm or type-II-dm or dm-type-2 or dm-type-II or NIDDM or IDDM or (insulin NEAR/3 (resistan* or sensitiv* or insensitiv*)) or fasting-insulin or homa-ir or homa2 or hyperinsulin* or prediabet* or (glucose NEAR/3 (intoleran* or toleran* or dysregulat*)) or igt or (glucose NEAR/3 (blood or plasma or level* or fasting or postprandial OR post-prandial)) OR "postprandial metabolism*" OR "post-prandial metabolism" or blood-sugar or hyperglycem* or hyperglycaem* or Hb-A1c or HbA1c or Hemoglobin-A1c or glycemi* or glycaemi* or area-under-the-curve or AUC or HOMA-IR or QUICKI or matsuda):ti,ab,kw | 1,945,041 |
| #2 | 'diet'/exp OR 'diet therapy'/exp OR 'caloric restriction'/exp OR 'feeding behavior'/exp OR 'fasting'/exp OR 'exercise'/exp OR 'kinesiotherapy'/exp OR 'sleep'/exp OR 'sleep deprivation'/exp OR 'circadian rhythm sleep disorder'/exp | 1,613,920 |
| #3 | 'time'/exp OR 'time factor'/exp OR 'sedentary time'/exp OR 'circadian rhythm'/exp OR 'circadian rhythm sleep disorder'/exp OR timing:ti,ab,kw OR schedul*:ti,ab,kw OR time*:ti,ab,kw OR morning:ti,ab,kw OR afternoon:ti,ab,kw OR evening:ti,ab,kw OR night:ti,ab,kw OR 'specific time':ti,ab,kw OR duration:ti,ab,kw OR exten*:ti,ab,kw OR intermittent:ti,ab,kw OR interval:ti,ab,kw OR periodic:ti,ab,kw OR 'alternate day':ti,ab,kw OR ((chrono* NEAR/3 nutrition):ti,ab,kw) OR chrononutrition:ti,ab,kw | 9,794,915 |
| #4 | ((diet OR nutrition* OR eat* OR food* OR feed* OR fasting OR 'energy restriction' OR 'caloric restriction' OR meal OR chrononutrition OR 'chrono nutrition' OR 'food intake' OR 'big breakfast' OR exercis* OR 'physic* activ*' OR workout OR train* OR run* OR swim* OR walk* OR sleep) NEAR/10 (timing OR schedul* OR time* OR morning OR afternoon OR evening OR night OR 'specific time' OR duration OR shift* OR exten* OR intermittent OR interval OR periodic OR 'alternate day')):ti,ab,kw | 471,220 |
| #5 | 'clinical trial'/exp OR 'clinical trial (topic)'/exp OR 'comparative study'/exp OR 'intervention'/exp OR 'health promotion'/exp OR (trial* OR rct OR random* OR ‘comparative study’ OR ‘laboratory protocol’ OR ‘controlled study’ OR crossover OR cross-over OR (compar* NEAR/2 group*) OR intervention* OR program* OR support OR advice OR counseling OR counselling OR training OR weight-management OR coaching OR motivational-interview* OR campaign*):ti,ab,kw | 9,306,399 |
| #6 | #2 AND #3 | 541,827 |
| #7 | #6 OR #4 | 812,189 |
| #8 | #1 AND #7 AND #5 | 50,990 |
| #9 | #8 NOT (('adolescent'/exp OR 'child'/exp OR adolescent*:ti,ab,kw,kw OR child*:ti,ab,kw OR schoolchild*:ti,ab,kw OR infant*:ti,ab,kw OR girl*:ti,ab,kw OR boy*:ti,ab,kw OR teen:ti,ab,kw OR teens:ti,ab,kw OR teenager*:ti,ab,kw OR youth*:ti,ab,kw OR pediatr*:ti,ab,kw OR paediatr*:ti,ab,kw OR puber*:ti,ab,kw ) NOT ('adult'/exp OR 'aged'/exp OR 'middle aged'/exp OR adult*:ti,ab,kw OR man:ti,ab,kw OR men:ti,ab,kw OR woman:ti,ab,kw OR women:ti,ab,kw)) | 48,092 |
| #10 | #9 NOT ('conference abstract'/it OR 'conference review'/it) | 32,597 |

S2: excluded articles per reason

| **Wrong intervention** |
| --- |
| 1. Astorino, T.A., et al., No effect of meal intake on physiological or perceptual responses to self-selected high intensity interval exercise (HIIE). Biol Sport, 2019. 36(3): p. 225-231. |
| 2. Benatti, F.B., et al., Intermittent Standing but not a Moderate Exercise Bout Reduces Postprandial Glycemia. Med Sci Sports Exerc, 2017. 49(11): p. 2305-2314. |
| 3. Blankenship, J.M., et al., Managing free-living hyperglycemia with exercise or interrupted sitting in type 2 diabetes. J Appl Physiol (1985), 2019. 126(3): p. 616-625. |
| 4. Chung, J., K. Kim, J. Hong, and H.J. Kong, Effects of prolonged exercise versus multiple short exercise sessions on risk for metabolic syndrome and the atherogenic index in middle-aged obese women: a randomised controlled trial. BMC Womens Health, 2017. 17(1): p. 65. |
| 5. Colberg, S.R., et al., Postprandial walking is better for lowering the glycemic effect of dinner than pre-dinner exercise in type 2 diabetic individuals. J Am Med Dir Assoc, 2009. 10(6): p. 394-7. |
| 6. De Jong, N.P., et al., Breaking up Sedentary Time in Overweight/Obese Adults on Work Days and Non-Work Days: Results from a Feasibility Study. Int J Environ Res Public Health, 2018. 15(11). |
| 7. Derave, W., et al., Effects of post-absorptive and postprandial exercise on glucoregulation in metabolic syndrome. Obesity (Silver Spring), 2007. 15(3): p. 704-11. |
| 8. Duvivier, B.M., et al., Breaking sitting with light activities vs structured exercise: a randomised crossover study demonstrating benefits for glycaemic control and insulin sensitivity in type 2 diabetes. Diabetologia, 2017. 60(3): p. 490-498. |
| 9. Edinburgh, R.M., et al., Preexercise breakfast ingestion versus extended overnight fasting increases postprandial glucose flux after exercise in healthy men. Am J Physiol Endocrinol Metab, 2018. 315(5): p. E1062-e1074. |
| 10. Erickson, M.L., et al., Postmeal exercise blunts postprandial glucose excursions in people on metformin monotherapy. J Appl Physiol (1985), 2017. 123(2): p. 444-450. |
| 11. Eriksen, L., I. Dahl-Petersen, S.B. Haugaard, and F. Dela, Comparison of the effect of multiple short-duration with single long-duration exercise sessions on glucose homeostasis in type 2 diabetes mellitus. Diabetologia, 2007. 50(11): p. 2245-53. |
| 12. Ferreira, A.P., et al., The influence of intense intermittent versus moderate continuous exercise on postprandial lipemia. Clinics (Sao Paulo), 2011. 66(4): p. 535-41. |
| 13. Gonzalez, J.T., R.C. Veasey, P.L. Rumbold, and E.J. Stevenson, Breakfast and exercise contingently affect postprandial metabolism and energy balance in physically active males. Br J Nutr, 2013. 110(4): p. 721-32. |
| 14. Goto, K., N. Ishii, A. Mizuno, and K. Takamatsu, Enhancement of fat metabolism by repeated bouts of moderate endurance exercise. J Appl Physiol (1985), 2007. 102(6): p. 2158-64. |
| 15. Hatamoto, Y., et al., Effect of exercise timing on elevated postprandial glucose levels. J Appl Physiol (1985), 2017. 123(2): p. 278-284. |
| 16. Heden, T.D., Y. Liu, and J.A. Kanaley, Exercise timing and blood lactate concentrations in individuals with type 2 diabetes. Appl Physiol Nutr Metab, 2017. 42(7): p. 732-737. |
| 17. Heden, T.D., et al., Postdinner resistance exercise improves postprandial risk factors more effectively than predinner resistance exercise in patients with type 2 diabetes. J Appl Physiol (1985), 2015. 118(5): p. 624-34. |
| 18. Hetherington-Rauth, M., et al., Morning versus afternoon physical activity and health-related outcomes in individuals with type 2 diabetes. Diabetes, Obesity and Metabolism, 2022. 24(6): p. 1172-1175. |
| 19. Karstoft, K., et al., The effects of free-living interval-walking training on glycemic control, body composition, and physical fitness in type 2 diabetic patients: a randomized, controlled trial. Diabetes Care, 2013. 36(2): p. 228-36. |
| 20. Kashiwabara, K., et al., Different Patterns of Walking and Postprandial Triglycerides in Older Women. Med Sci Sports Exerc, 2018. 50(1): p. 79-87. |
| 21. Katsanos, C.S. and R.J. Moffatt, Acute effects of premeal versus postmeal exercise on postprandial hypertriglyceridemia. Clin J Sport Med, 2004. 14(1): p. 33-9. |
| 22. Koenigstorfer, J. and W.F. Schmidt, Effects of exercise training and a hypocaloric diet on female monozygotic twins in free-living conditions. Physiol Behav, 2011. 104(5): p. 838-44. |
| 23. Larsen, R.N., et al., Does the type of activity "break" from prolonged sitting differentially impact on postprandial blood glucose reductions? An exploratory analysis. Appl Physiol Nutr Metab, 2017. 42(8): p. 897-900. |
| 24. Mackie, P., et al., Acute Effects of Frequent Light-Intensity Standing-Based Exercises That Interrupt 8 Hours of Prolonged Sitting on Postprandial Glucose in Stroke Survivors: A Dose-Escalation Trial. J Phys Act Health, 2021. 18(6): p. 644-652. |
| 25. Maylor, B.D., J.K. Zakrzewski-Fruer, C.J. Orton, and D.P. Bailey, Beneficial postprandial lipaemic effects of interrupting sedentary time with high-intensity physical activity versus a continuous moderate-intensity physical activity bout: A randomised crossover trial. J Sci Med Sport, 2018. 21(12): p. 1250-1255. |
| 26. Maylor, B.D., et al., Effects of Frequency and Duration of Interrupting Sitting on Cardiometabolic Risk Markers. Int J Sports Med, 2019. 40(13): p. 818-824. |
| 27. McIver, V.J., L. Mattin, G.H. Evans, and A.M.W. Yau, The effect of brisk walking in the fasted versus fed state on metabolic responses, gastrointestinal function, and appetite in healthy men. Int J Obes (Lond), 2019. 43(9): p. 1691-1700. |
| 28. Miyashita, M., et al., Interrupting Sitting Time with Regular Walks Attenuates Postprandial Triglycerides. Int J Sports Med, 2016. 37(2): p. 97-103. |
| 29. Murphy, M.H., A.M. Nevill, and A.E. Hardman, Different patterns of brisk walking are equally effective in decreasing postprandial lipaemia. Int J Obes Relat Metab Disord, 2000. 24(10): p. 1303-9. |
| 30. Nygaard, H., et al., Acute effects of post-absorptive and postprandial moderate exercise on markers of inflammation in hyperglycemic individuals. Eur J Appl Physiol, 2017. 117(4): p. 787-794. |
| 31. Nygaard, H., et al., Effects of Exercise in the Fasted and Postprandial State on Interstitial Glucose in Hyperglycemic Individuals. J Sports Sci Med, 2017. 16(2): p. 254-263. |
| 32. Pahra, D., et al., Impact of post-meal and one-time daily exercise in patient with type 2 diabetes mellitus: a randomized crossover study. Diabetol Metab Syndr, 2017. 9: p. 64. |
| 33. Peddie, M.C., et al., Breaking prolonged sitting reduces postprandial glycemia in healthy, normal-weight adults: a randomized crossover trial. Am J Clin Nutr, 2013. 98(2): p. 358-66. |
| 34. Poirier, P., et al., Prior meal enhances the plasma glucose lowering effect of exercise in type 2 diabetes. Med Sci Sports Exerc, 2001. 33(8): p. 1259-64. |
| 35. Price, M. and K. Halabi, The effects of work-rest duration on intermittent exercise and subsequent performance. J Sports Sci, 2005. 23(8): p. 835-42. |
| 36. Price, M. and P. Moss, The effects of work:rest duration on physiological and perceptual responses during intermittent exercise and performance. J Sports Sci, 2007. 25(14): p. 1613-21. |
| 37. Reynolds, A.N., J.I. Mann, S. Williams, and B.J. Venn, Advice to walk after meals is more effective for lowering postprandial glycaemia in type 2 diabetes mellitus than advice that does not specify timing: a randomised crossover study. Diabetologia, 2016. 59(12): p. 2572-2578. |
| 38. Savikj, M., et al., Afternoon exercise is more efficacious than morning exercise at improving blood glucose levels in individuals with type 2 diabetes: a randomised crossover trial. Diabetologia, 2019. 62(2): p. 233-237. |
| 39. Savikj, M., et al., Exercise timing influences multi-tissue metabolome and skeletal muscle proteome profiles in type 2 diabetic patients – A randomized crossover trial. Metabolism, 2022. 135: p. 155268. |
| 40. Schabort, E.J., A.N. Bosch, S.M. Weltan, and T.D. Noakes, The effect of a preexercise meal on time to fatigue during prolonged cycling exercise. Med Sci Sports Exerc, 1999. 31(3): p. 464-71. |
| 41. Solomon, T.P.J., et al., Immediate post-breakfast physical activity improves interstitial postprandial glycemia: a comparison of different activity-meal timings. Pflugers Arch, 2020. 472(2): p. 271-280. |
| 42. Toghi-Eshghi, S.R. and J.E. Yardley, Morning (Fasting) vs Afternoon Resistance Exercise in Individuals With Type 1 Diabetes: A Randomized Crossover Study. The Journal of Clinical Endocrinology & Metabolism, 2019. 104(11): p. 5217-5224. |
| 43. Van Proeyen, K., et al., Beneficial metabolic adaptations due to endurance exercise training in the fasted state. J Appl Physiol (1985), 2011. 110(1): p. 236-45. |
| 44. Wheeler, M.J., et al., Combined effects of continuous exercise and intermittent active interruptions to prolonged sitting on postprandial glucose, insulin, and triglycerides in adults with obesity: a randomized crossover trial. Int J Behav Nutr Phys Act, 2020. 17(1): p. 152. |
| 45. Whyte, L.J., et al., Effects of single bout of very high-intensity exercise on metabolic health biomarkers in overweight/obese sedentary men. Metabolism, 2013. 62(2): p. 212-9. |
| 46. Yamanouchi, K., et al., The effect of walking before and after breakfast on blood glucose levels in patients with type 1 diabetes treated with intensive insulin therapy. Diabetes Res Clin Pract, 2002. 58(1): p. 11-8. |
| 47. Yap, M.C., G. Balasekaran, and S.F. Burns, Acute effect of 30 min of accumulated versus continuous brisk walking on insulin sensitivity in young Asian adults. Eur J Appl Physiol, 2015. 115(9): p. 1867-75. |
| 48. Yardley, J.E., Fasting May Alter Blood Glucose Responses to High-Intensity Interval Exercise in Adults With Type 1 Diabetes: A Randomized, Acute Crossover Study. Can J Diabetes, 2020. 44(8): p. 727-733. |
| 49. Zhang, J.Q., et al., Effect of exercise timing on postprandial lipemia in hypertriglyceridemic men. Can J Appl Physiol, 2004. 29(5): p. 590-603. |
|  |
| **Wrong outcome** |
| 50. Jusup, S., et al., Morning Exercise is More Effective in Ameliorating Oxidative Stress in Patients with Type 2 Diabetes Mellitus. Open Access Macedonian Journal of Medical Sciences, 2022. 10(A): p. 1499-1504. |
| 51. Teo, S.Y.M., et al., Effects of diurnal exercise timing on appetite, energy intake and body composition: A parallel randomized trial. Appetite, 2021. 167: p. 105600. |
| 52. Thomas, J.M., et al., Circadian rhythm phase shifts caused by timed exercise vary with chronotype. JCI Insight, 2020. 5(3). |
| 53. Wheeler, M.J., et al., Distinct effects of acute exercise and breaks in sitting on working memory and executive function in older adults: a three-arm, randomised cross-over trial to evaluate the effects of exercise with and without breaks in sitting on cognition. Br J Sports Med, 2020. 54(13): p. 776-781. |
| 54. Zhang, J.Q., T.R. Thomas, and S.D. Ball, Effect of exercise timing on postprandial lipemia and HDL cholesterol subfractions. J Appl Physiol (1985), 1998. 85(4): p. 1516-22. |
| 55. Taylor, F.C., et al., Acute effects of interrupting prolonged sitting on vascular function in type 2 diabetes. Am J Physiol Heart Circ Physiol, 2021. 320(1): p. H393-h403. |
|  |
| **Full text unavailable** |
| 56. Alizadeh, Z., R. Kordi, M.J.H.Z. Attar, and M.A. Mansournia, The effects of continuous and intermittent aerobic exercise on lipid profile and fasting blood sugar in women with a body mass index more than 25 kg/m2: A randomized controlled trial. Tehran University Medical Journal, 2011. 69(4): p. 253-259. |
| 57. Darvakh, H. and A. Mousavian, Effect of 12 Weeks Aerobic Exercise in morning and afternoon on Diabetic Women’s protein Catabolism. The Iranian Journal of Obstetrics, Gynecology and Infertility, 2017. 20(2): p. 60-67. |
| 58. Li, Z., Y. Hu, and J. Ma, [Effect of moderate exercise for 30 min at 30 min versus 60 min after dinner on glycemic control in patients with type 2 diabetes: a randomized, crossover, self-controlled study]. Nan Fang Yi Ke Da Xue Xue Bao, 2018. 38(10): p. 1165-1170. |
|  |
| **Wrong language** |
| 59. Cardoso, S.S., et al., Chronobiology of exercise: the influence of scheduling upon glycemic responses of control and of subjects with diabetes mellitus. Prog Clin Biol Res, 1990. 341b: p. 345-53. |
| 60. Freire, Y.A., et al., Effect of Breaks in Prolonged Sitting or Low-Volume High-Intensity Interval Exercise on Markers of Metabolic Syndrome in Adults With Excess Body Fat: A Crossover Trial. J Phys Act Health, 2019. 16(9): p. 727-735. |
| 61. Weydahl, A. and R.B. Sothern, Glycemic Response to Exercise in the Subarctic Depends on Time of Day, Season and Sex. Biological Rhythm Research, 1997. 28(1): p. 42-55. |
|  |
| **Duplicate** |
| 62. Li, T.L. and M. Gleeson, The effect of single and repeated bouts of prolonged cycling and circadian variation on saliva flow rate, immunoglobulin A and alpha-amylase responses. J Sports Sci, 2004. 22(11-12): p. 1015-24. |

S3: Forestplots


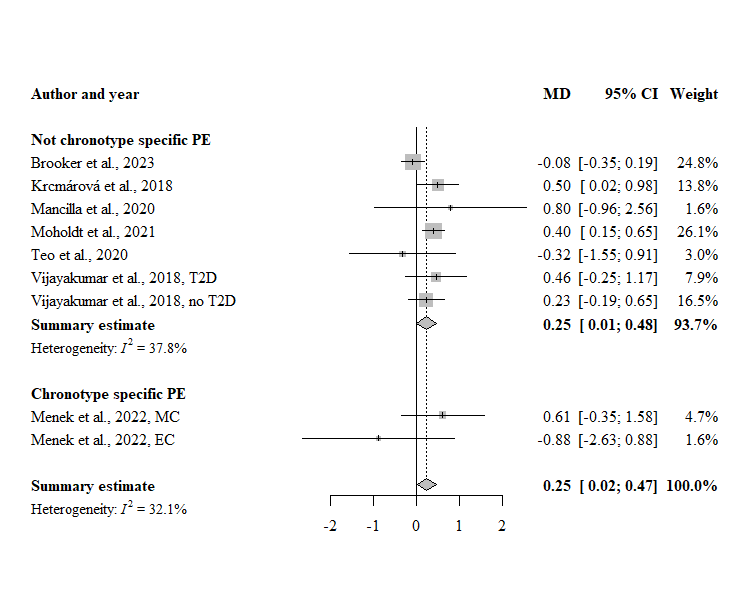


S3.1: Forest plot fasting blood glucose long-term studies, excluding Menek et al. Data presented as pooled mean difference (MD; mmol/L); 95%CI: 95% Confidence Interval; EC: evening chronotype; MC = morning chronotype; T2D: type 2 diabetes.


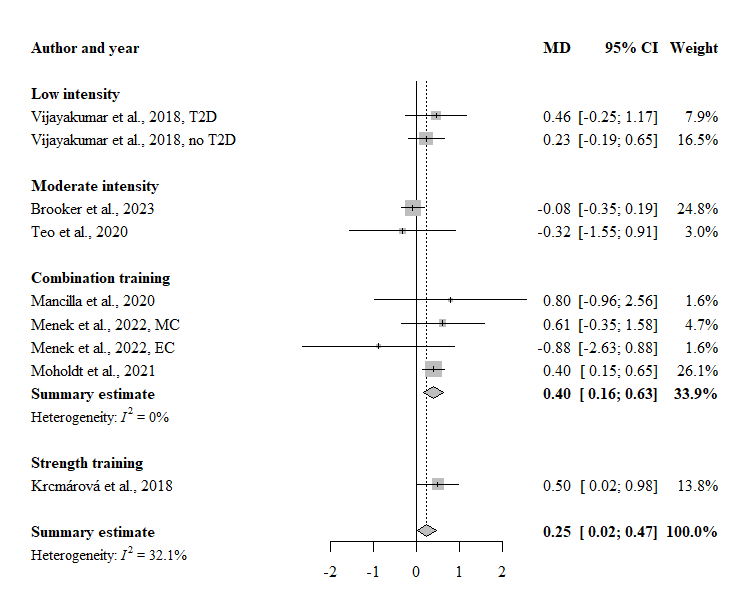


S3.2: forest plot fasting blood glucose long-term studies, stratified by training intensity. Data presented as pooled mean difference (MD; mmol/L); 95%CI: 95% Confidence Interval; EC: evening chronotype; MC: morning chronotype; PE: physical exercise; T2D: type 2 diabetes


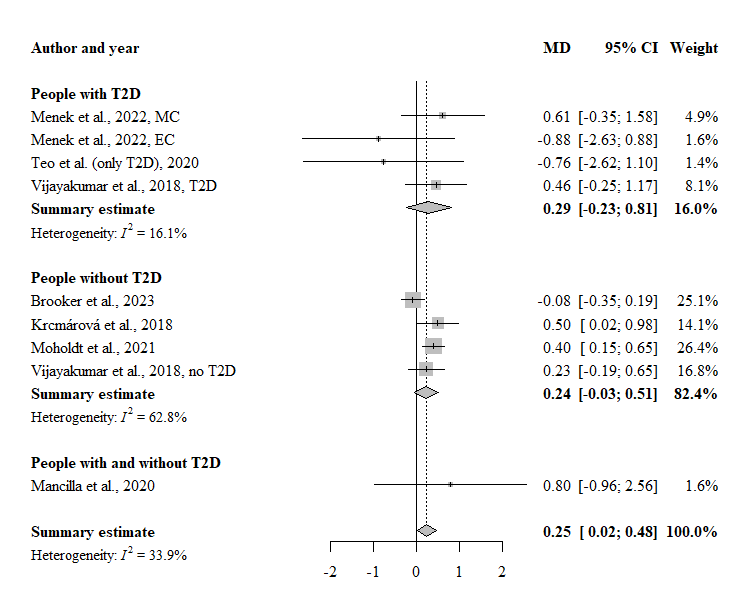


S3.3: forest plot fasting blood glucose long-term studies, stratified by health status. Data presented as pooled mean difference (MD; mmol/L); 95%CI: 95% Confidence Interval; EC: evening chronotype; MC: morning chronotype; PE: physical exercise; T2D: type 2 diabetes


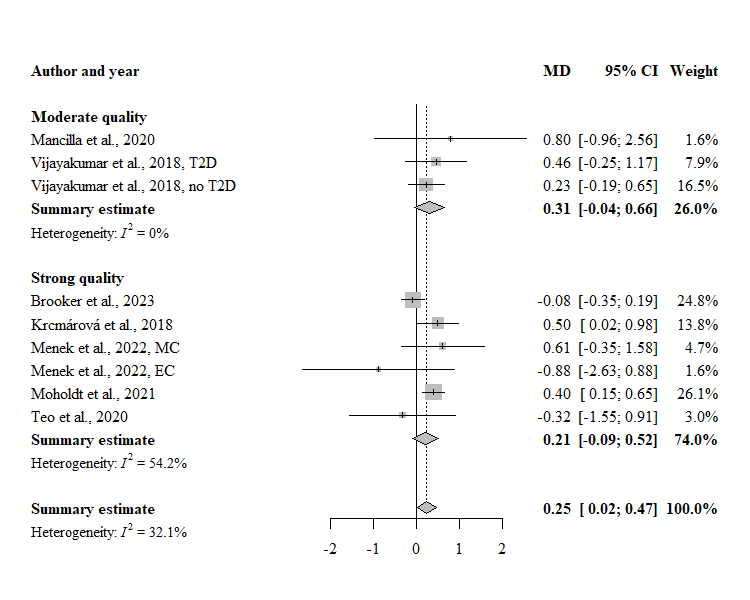


S3.4: forest plot fasting blood glucose long-term studies, stratified by study quality. Data presented as pooled mean difference (MD; mmol/L); 95%CI: 95% Confidence Interval; EC: evening chronotype; MC: morning chronotype; PE: physical exercise; T2D: type 2 diabetes

S4: Funnel plots


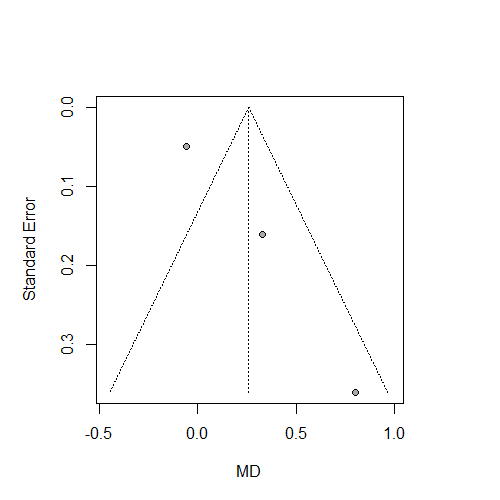


S4.1: Funnel plot acute studies glucose directly after physical exercise (p=0.0779)


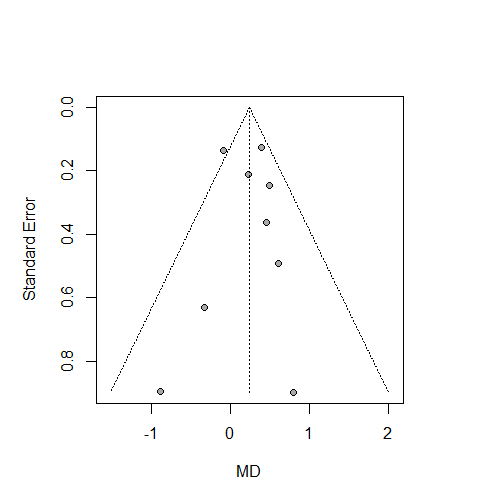


S4.2: Funnel plot long-term studies fasting glucose (p=0.9418)
